# When European meets African honeybees (*Apis mellifera* L.) in the tropics: Morphological changes related to genetics in Mauritius Island (South-West Indian Ocean)

**DOI:** 10.1371/journal.pone.0242053

**Published:** 2020-11-19

**Authors:** Julien Galataud, Hélène Delatte, Maéva Angélique Techer, Christophe Simiand, Preeaduth Sookar, Bernard Reynaud, Johanna Clémencet

**Affiliations:** 1 Université de La Réunion, UMR PVBMT, La Réunion, France; 2 CIRAD, UMR PVBMT, 7 chemin de l’Irat, Ligne Paradis, Saint Pierre, La Réunion, France; 3 Okinawa Institute of Science and Technology, Okinawa, Japan; 4 Ministry of Agro Industry and Food Security, Agricultural Services, Reduit, Mauritius; 5 Université de La Réunion, UMR PVBMT, Saint Pierre, La Réunion, France; University of Iceland, ICELAND

## Abstract

The previous genetic characterization of the honeybee population of Mauritius Island (Indian Ocean) revealed an ongoing process of hybridization between the first established African subspecies *Apis mellifera unicolor* and recently imported European subspecies (*A*. *m*. *ligustica*, *A*. *m*. *carnica* and *A*. *m*. *mellifera*). This context offers the rare opportunity to explore the influence of hybridization between African and European honeybees on phenotypic traits out of the case largely studied of the Africanized honeybee (hybrid between *A*. *m*. *scutellata* from South Africa and European subspecies). We thus conducted geometric morphometric analyses on forewings of 283 workers genetically characterized at 14 microsatellite loci to evaluate (1) if the morphological variability coincides well with the neutral genetic variability, (2) if hybrids exhibited rather parental, intermediate or transgressive traits, and (3) to test if fluctuating asymmetry (FA) of size and shape, as a measure of developmental stability, was elevated in hybrids (due to genetic stress) and/or European bees (due to unsuitable environment) compared to African bees. A strong concordance was found between morphological variability and neutral genetic variability, especially for wing shape, based on partial least-square analyses (PLS). However, on average, the morphology of hybrids was more similar to the African bees, potentially reflecting the dynamics and direction of introgression. Significant FA for wing size as well as wing shape was detected, suggesting the overall presence of stress during the development of the studied individuals. In contrast, the asymmetry levels do not differ according to the ancestry (African, European or hybrid) of the individuals. Therefore, if ongoing hybridization contributed to increasing the genetic and phenotypic diversity of the populations and influences its adaptive potential, developmental stressors could not be identified and their evolutionary consequences remain uncertain.

## Introduction

Hybridization, defined as the contact between previously isolated lineages that result in viable and—to a varying degree—fertile offspring [1], has been extensively facilitated by human movements and activities, which intensify and ignore natural boundaries [2, 3]. The recent establishment of gene flows through the dissemination—voluntary or not—of individuals out of their native range may result in increased phenotypic variation, especially in closely related populations with weak or non-existent reproductive barriers. Increased phenotypic variation is typically observed among individuals as a consequence of augmented genetic variability. The import of new alleles and the consequent novel genomic combinations formed by admixture and repeated backcrossings can generate a variety of phenotypes in the hybrid offspring. These can range from parental types to intermediate or transgressive (extreme) forms, depending on both gene interactions (e.g. additive effects, over-dominance, epistasis), environmental influences on gene expression (external factors, e.g. temperatures, or internal factors, e.g. metabolism), and the relative contribution of natural selection and stochastic processes (dispersal and drift). Inbreeding and exposure to unfamiliar environmental conditions may also affect the ability of individuals to undergo stable development, through disruption of coadapted gene complexes in hybrid offspring, and as a consequence of radical environmental changes for imported genomes [4, 5]. Development instability (DI) refers to slight and random deviations to perfect left-right symmetry in structures, and is classically measured as fluctuating asymmetry (FA). In different taxa, excess of FA could be associated with (human-mediated) genetic [6–10] and environmental [11–14] stresses, and reduced adaptive value or fitness of individuals [15–17]. Thus, relating both components of morphological variation, i.e. between and within individuals variation (*via* FA), with neutral genetic markers permits hybrid characterization, and may provide valuable insight on the extent of hybridization as well as the mechanisms shaping hybrid populations.

The biological model we used to address these questions is the Western honeybee *Apis mellifera*, whose distribution is nowadays much influenced by beekeeping activities [18]. Four major evolutionary lineages occupying a wide natural range have been described by combining morphological and molecular approaches (A: African lineage, C and M: West and South-East European lineages, O: Middle East lineage) [19–23]. Nearly thirty *A*. *mellifera* subspecies and various ecotypes were identified and can supposedly all interbreed due to absent or weak reproductive isolating barriers [24–26]. The highly polyandrous mating system of the honeybee with its extensive mating flights [27], coupled with large-scale migratory beekeeping and trade-in queens, promoted gene flow and hybridization between formerly isolated honeybee populations within and outside its natural range [18]. The Africanized honeybee (AHB), a hybrid between *Apis mellifera scutellata* (native of Africa) and European-derived strains, which spread across America in a few decades and supplanted the pre-established European colonies, illustrates dramatically how human-induced hybridization can promote diversification and colonization success. The genome of the AHB is characterized by a large predominance of African ancestry [28–31], most likely due to a predisposition of African traits to South American environmental conditions and genetic and behavioral biases that benefit African alleles [32]. In addition, a positive selection for Europeans-derived alleles has also been highlighted in part of the genome [31]. The AHB therefore owes its success to the human-initiated hybridization, pre-adaptations to tropical and subtropical climate, and the adaptive exploitation of imported diversity. Among the traits that characterize the AHB, the size and shape of the wings have proven to be discriminating enough to identify it quickly and efficiently [32–35]. Moreover, wing shape of African worker bees would show greater developmental stability, which could contribute to asymmetrical gene flow and the prevalence of African ancestry if related to better fitness (e.g., better flight skills) [36]. In short, the evolutionary trajectory of the American honeybee took a whole new direction following hybridization, and the study of both genetic and phenotypic markers helps to better understanding the mechanisms underlying this evolutionary shift.

In this study we focused on the honeybee population of Mauritius Island, located in the South-West Indian Ocean, which also experiences ongoing hybridization between African and European lineages. It offers the opportunity to study the effects of relatively recent hybridization on traits variability in tropical and insular environmental conditions. Latest genetic studies confirmed that the initial honeybee population of Mauritius belongs to the African lineage, closely related to *A*. *m*. *unicolor* [37] subspecies, endemic of Madagascar and established in surrounding archipelagos [38–40]. Mitochondrial signatures of repeated importations of European continental subspecies in the current population have been detected, and the analyses of genetic diversity revealed the occurrence of two hybridizing gene pools, with European and African ancestries sampled in near-balanced proportions (about 55% of A lineage, 44% of European C lineage, 1% of European M lineage) [39]. In this paper, morphological variability of Mauritius honeybees was examined on forewings size and venation patterns of the worker honeybees *via* geometric morphometric methods. Geo-morphometrics are now commonly used in insect micro-taxonomy, and have been proved very effective in the identification of honeybee subspecies, ecotypes and hybrids [20, 21, 23, 41]. We then related between and among individuals variation of morphometric traits with neutral genetic structuring and the degree of hybridization of the individuals previously inferred from Bayesian analysis of microsatellite markers [40]. The relation between hybridization on developmental instabilities, i.e. individual FA, was also examined on both forewing size and shape.

## Materials and methods

Recent genetic work by Técher et al. [39, 40] has shown that the population of Mauritius consists of two hybridizing genetic clusters: one African related to *A*. *m*. *unicolor*, whose origin (natural or anthropogenic) is not fully elucidated, and another European from successive imports. In order to better depict the honeybee population of Mauritius, the same bees were used for this study. They consist of workers sampled in 2012 in apiaries with 1 to 150 colonies, all managed by humans except for one wild colony. The bees were collected at the entrance or inside the hives, depending on the site. All of this information is compiled in Table 1.

**Table 1:** Genetic categorization and contextual data on honeybees (*Apis mellifera*) sampled in Mauritius.

Site	Maternal lineage (mtDNA)			Hybrid index (nDNA)				*N*	Date	*N* colonies/apiary	Type	Sampling	Location			
	A	C	M	Afr.	Hybrid	Eur.	na						Latitude	Longitude	GPS *X*	GPS *Y*
**MUS01**		1			1			**1**	14/11/12	1	M	E	S 20°23'00.1"	EO 57°25'16.4"	-20.38333	57.42111
**MUS02**	19			12	7			**19**	14/11/12	20	M	E	S 20°19'55.4"	EO 57°31'17.5"	-20.33194	57.52139
**MUS03**		1			1			**1**	13/11/12	1	M	E	S 20°11'45.5"	EO 57°35'34.6"	-20.19583	57.59278
**MUS04**	9			4	4		1	**9**	13/11/12	16	M	E	S 20°12'58.5"	EO 57°32'20.4"	-20.21611	57.53889
**MUS05**	1	2		1	2			**3**	13/11/12	3	M	E	S 20°14'00.0"	EO 57°29'24.9"	-20.23333	57.49
**MUS06**	9			5	3		1	**9**	15/11/12	10	M	E	S 20°14'33.0"	EO 57°28'38.0"	-20.242451	57.477089
**MUS07**	12	1		3	9		1	**13**	15/11/12	13	M	E	S 20°14'20.7"	EO 57°27'04.1"	-20.23889	57.45111
**MUS08**	14	1	2	5	12			**17**	14/11/12	22	M	I	S 20°12'06.4"	EO 57°25'12.0"	-20.20167	57.42
**MUS09**	6	16	2	3	17	2	2	**24**	15/11/12	30	M	E	S 20°10'10.7"	EO 57°27'17.2"	-20.16944	57.45472
**MUS12**	19	1		9	10		1	**20**	13/11/12	23	M	E	S 20°06'00.9"	EO 57°32'27.4"	-20.1	57.54083
**MUS13**		3			3			**3**	12/11/12	4	M	E	S 20°06'10.5"	EO 57°34'54.3"	-20.10278	57.58167
**MUS14**	1			1				**1**	15/11/12	1	W	I	S 20°06'18.2"	EO 57°38'56.7"	-20.105	57.64889
**MUS15**	4	4		3	5			**8**	15/11/12	8	M	E	S 20°04'19.5"	EO 57°40'26.7"	-20.07194	57.67389
**MUS16**	13			8	4		1	**13**	12/11/12	15	M	E	S 20°07'00.5"	EO 57°42'03.3"	-20.11667	57.70083
**MUS17**	13	1		3	10		1	**14**	15/11/12	15	M	E	S 20°06'38.8"	EO 57°43'08.4"	-20.11056	57.71889
**MUS18**	21	1		11	11			**22**	16/11/12	45	M	I	S 20°07'58.5"	EO 57°43'09.5"	-20.13278	57.71917
**MUS19**	4	9		2	10	1		**13**	16/11/12	19	M	E	S 20°08'17.6"	EO 57°44'41.1"	-20.13806	57.74472
**MUS20**		70			48	17	5	**70**	12/11/12	150	M	E	S 20°08'56.3"	EO 57°43'35.3"	-20.14889	57.72639
**MUS21**	1	18			11	6	2	**19**	12/11/12	21	M	E	S 20°11'40.1"	EO 57°43'38.7"	-20.19444	57.72722
**MUS22**	1			1				**1**	13/11/12	1	M	E	S 20°14'53.3"	EO 57°40'52.9"	-20.24806	57.68111
**MUS23**	1			1				**1**	13/11/12	1	M	E	S 20°17'17.2"	EO 57°41'32.2"	-20.28806	57.69222
**MUS24**	2			1	1			**2**	16/11/12	2	M	E	S 20°25'53.2"	EO 57°39'43.4"	-20.43139	57.66194
**Total**	**150**	**129**	**4**	**73**	**169**	**26**	**15**	**283**					**–**	**–**	**–**	**–**

MUS01-24, site as designated in [40]; mtDNA, mitochondrial DNA (COI-COII intergenic region); A, African lineage; C, Eastern European lineage; M, Western European lineage; nDNA, nuclear DNA; Hybrid index (HI), Bayesian estimates of individuals’ admixture (see Techer et al. [40]) subdivided into three discrete categories; Afr., “pure” Africans (HI ≤ 0.1); Hybrids (0.1 < HI < 0.9); Eur., “pure” Europeans (HI ≥ 0.9) [59]; na, data not available; *N*, number of individuals; M, managed colony; W, wild colony; E, entrance; I; inside.

### Genetic data

The use of mitochondrial markers (length and sequence of the COI-COII intergenic region [20, 42, 43]) allowed Técher et al. [39] to identify the maternal origin of each individual. Three evolutionary lineages are thus represented in the sample (Table 1): the African lineage A grouping the African subspecies, the European lineage C including subspecies naturally distributed east and south of the Alps, and the European lineage M including subspecies distributed in north and west of Europe [19]. On the other hand, a Bayesian approach involving 14 microsatellite loci (A113, A24, AP55, A88, A28, A29, AP289, AP273, (A) B124, A8, A35, AP33, AP66, and AP43) [40, 44] permitted to estimate the degree of hybridization of the individuals. This translates into a hybrid index (HI), which ranges from 0 for African ancestry to 1 for European ancestry. Intermediate values reflect hybridization to varying degrees. For convenience, HI was used as a continuous variable or transformed into a discrete variable, depending on the hypothesis tested (see Table 1 and analyzes below). The raw data containing the allele frequencies of each microsatellite marker for each individual was also retrieved for analyzes.

### Wing data acquisition

**Fig 1:**
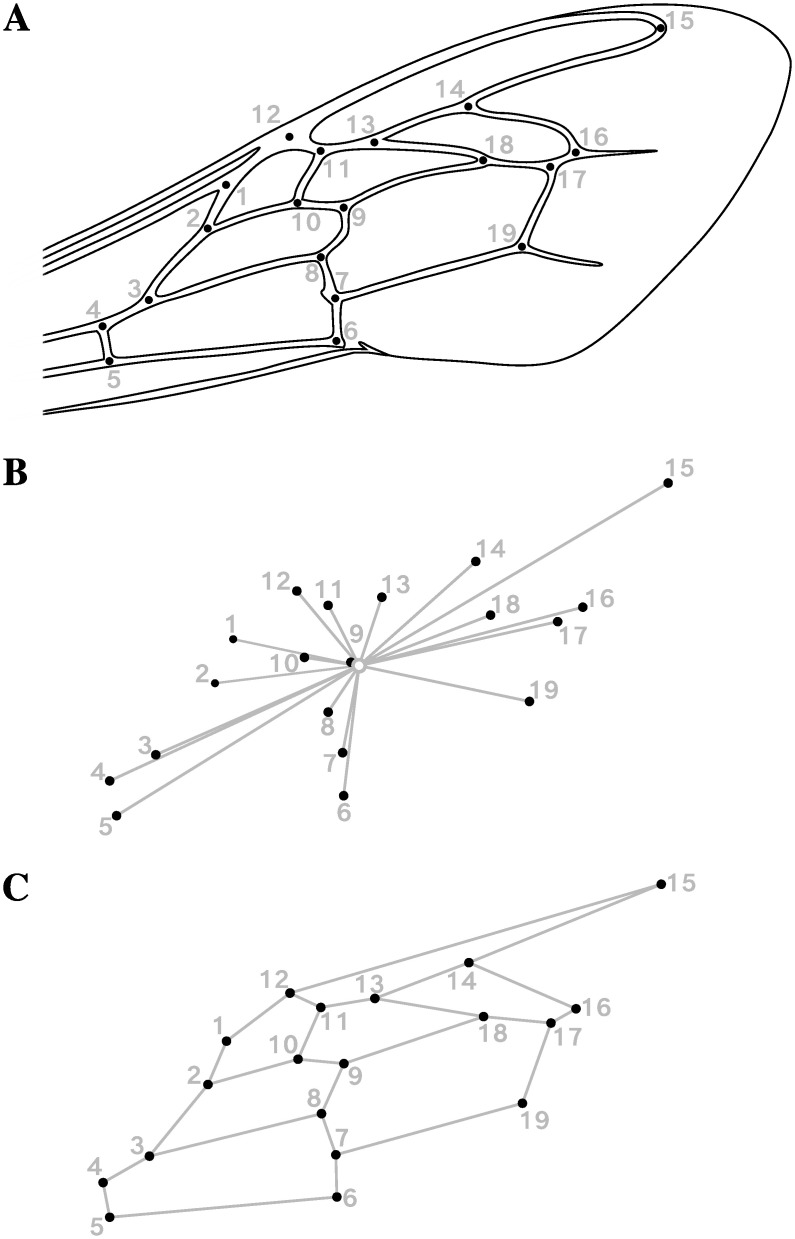
Geometric morphometrics of the forewing venation of honeybee workers (*Apis mellifera*). (A) Location of the 19 landmarks digitized on the right and (mirrored) left forewings of *Apis mellifera* workers, following Smith et al. [45] procedure. (B) Visual representation of the centroid size as computed for 19 landmarks on a forewing of *Apis mellifera*. The open circle is the centroid (i.e. the barycenter) of the landmark configuration; the segments connecting the centroid to the landmarks represent the distances used to compute centroid size. (C) Wireframe visualization of forewing shape. The landmarks are connected with straight lines depicting the wing venation pattern.

The above-mentioned genetic characterizations were carried out using DNA extracted from the legs of the bees. All remaining biological material was stored in 90% ethanol at -20 ° C. Under these conditions, the wings, when not damaged by capture or manipulations, kept their venation pattern intact. Thus, only individuals whose right and left wings were available and correctly preserved were used to perform morphometric measurements. For this, the wings of each individual were cut at their basis, mounted in distilled water on a micrometric slide for scaling, and then photographed with a high-resolution camera mounted on a microscope (Leica DMC5400, Leica Microsystems). Nineteen landmarks were digitized at the vein intersections following Smith et al. [45] procedure (Fig 1A). The x and y Cartesian coordinates of each landmark were collected in ImageJ version 1.49u [46] with the Point Picker plugin (http://bigwww.epfl.ch/thevenaz/pointpicker/) [47]. To avoid any user effect, all measurements were made by a single operator. A total of 283 individuals already genetically characterized (lineage, allele frequencies, HI) were thus characterized morphologically. In addition, 36 individuals randomly selected were photographed and digitized twice in separate sessions to quantify measurement errors related to image acquisition and landmark positioning.

### Forewing size and shape extraction

Overall wing size was estimated by the centroid size (CS), a measure equal to the square root of the sum of the squared distances between each landmark and the centroid, i.e. the barycenter of the landmark configuration (Fig 1B). Wing shape variables were extracted from x and y coordinates of the 19 landmarks *via* a generalized least-squares Procrustes superimposition with the following steps [48, 49] (Fig 1C): *(1)* right and—previously mirrored—left wings of each individual were centered on their centroid, then *(2)* normalized to unit centroid-size (CS = 1.0) to remove size and shape association (i.e. isometry), and *(3)* iteratively rotated to minimize the sum of squared distances between each configuration and the sample mean. Thus, any difference in Procrustes coordinates of corresponding landmarks between two configurations resulted from (isometry-free) shape differences, expressed in units of Procrustes distances (dimensionless). As a consequence of Procrustes superimposition steps, 4 degrees of freedom are lost (two for centering, one for scaling, and one for rotation) resulting in 38–4 = 34 shape space dimensions.

### Amounts of among individuals’ variation and asymmetry

The first step consisted in testing the presence of directional and fluctuating asymmetry while accounting for measurement errors relating to both the position and orientation of the wings in the photos and the digitization of the landmarks at vein intersections. An ANOVA on CS for size [50, 51] and a Procrustes ANOVA on Procrustes distances for shape [49, 52] were therefore conducted on the subsample of 36 individuals whose forewings were photographed and digitized twice. Both analyses follow the same two-way mixed model design and partition the total variation into components of symmetric and asymmetric variation. Following this design, the main random effect ‘individual’ represents the variation among individuals and is computed as individuals’ left-right means (corrected for any type of asymmetry). The second main and fixed effect ‘side’ represents directional asymmetry (DA) and is computed as mean of left-right differences. Fluctuating asymmetry (FA), expressed as individual left-right differences, is represented by the interaction term ‘individual × side’ (a mixed effect). Measurement errors due to imaging (ME1) and digitizing (ME2) are quantified by the variation over replicates for each individual and side. Statistical significance of the various effects was assessed with parametric Goodall’s *F* tests [53], based on summed square Procrustes distances in the case of shape [51]. Ultimately, to determine the proportion of size or shape variation explained by each factor in the whole sample, the same analyzes were conducted on the complete data but in the absence of replicates, after proving the measurement errors as negligible (see Results). All the analyses mentioned above were conducted with the software MorphoJ (https://www.R-project.org/) [54].

### Congruence between morphological and neutral genetic variation

To assess the congruence between morphometric and genetic datasets, we conducted partial least squares (PLS) analyses in MorphoJ using alternatively wings size and shape (i.e., centroid sizes and Procrustes coordinates) against microsatellite allele frequencies. The PLS method is based on a singular value decomposition of the matrix of covariances between two separate blocks of variables [55, 56]. It resulted in paired axes uncorrelated and ordered by decreasing singular values, each forming linear combinations of the variables from each dataset and accounting for as much as possible of the overall covariance. The number of axes was equal to the number of dimensions in the smaller dataset (i.e. 1 for size and 34 for shape). The overall association between morphological and genetic datasets was assessed by the Escoufier’s RV coefficient [57], a multivariate generalization of the squared Pearson correlation coefficient (*r*). Singular values, correlations between paired PLS axes as well as RV coefficients were statistically tested by randomly permuting the individuals (rows) in the shape block (10,000 iterations), so that the covariance structure between blocks was modified without altering the variance within blocks [55, 56]. Additionally, to parallel dominant patterns of covariance between blocks to the major patterns of variation within each block, PLS singular axes were compared to their counterparts from principal component analyses conducted separately on shape and genetic datasets. For this purpose, angles and correlations between corresponding PLS and PCA axes were computed and statistically tested in MorphoJ [58]. Regarding genetic data, the results of multivariate analyses were faced with the Bayesian clustering method by measuring correlation of PLS and PCA scores with hybrid index values.

### Effects of hybridization on wing morphology

The hybrid index (HI) was directly exploited to specifically address how the current intraspecific admixture between two genetic pools, one prior African related to *A*. *m*. *unicolor* and one European composed of recently imported subspecies (C and M lineages), affected wings size and shape of honeybees in Mauritius. As a first step, HI was used as a grouping variable to distinct hybridized individuals from non-hybridized. Three clusters were created following Vähä and Primmer [59] recommendations: the workers were classified as ‘pure’ Africans if HI was smaller than 0.1 (*N*_AFR_ = 74), as ‘pure’ Europeans if HI was larger than 0.9 (*N*_EUR_ = 27), and as hybrids (*N*_H_ = 177) if HI was contained between those values [22, 60]. This results in means/medians values of 0.06/0.06 for ‘pure’ Africans, 0.39/0.32 for hybrids, and 0.96/0.97 for ‘pure’ Europeans, denoting that hybrid values are slightly shifted towards the African genetic cluster. A one-way ANOVA with post-hoc tests was conducted to compare group means based on wings size. Regarding wings shape, given the unequal distribution of individuals in different clusters and the high dimensionality of the Procrustes dataset (34 dimensions), a between-group PCA was conducted, as recommended by Mitteroecker and Bookstein [61]. This allowed (*1*) examining the relative arrangement of the three clusters in a reduced space of two dimensions, (*2*) evaluating the significance of group separation, and (*3*) visualizing related shape changes. Pairwise mean differences and reliability of classification rates were assessed by a leave-one-out cross-validation and permutation tests (30,000 iterations). To determine if hybrids presented phenotypic transgression, the angle formed by the vectors joining the cluster means (Hybrids—Africans—Europeans: $\hat{HAE}$) in the shape-space was computed and tested. This analyze was achieved in RStudio software (https://www.R-project.org/) [62], using the trajectory.analysis function from the package geomorph [63, 64]. Additionally, correlations between size, bgPC1 and bgPC2 scores and HI were estimated.

### Effect of hybridization on wing asymmetry

Size FA (fluctuating asymmetry) of each individual was given by the difference between the CS of the left and the right forewing. The amount of shape FA of each individual was synthetized in the form of univariate scores based on either Procrustes distances (Procrustes FA scores) or Mahalanobis distances (Mahalanobis FA scores), which take the covariance structure into account [56, 65]. To establish the reliability of these measures (delivered in MorphoJ when performing ANOVAs), intraclass correlation coefficients (2,1) were computed in the sub-dataset with repeats [66, 67]. The association between the amounts of FA and size variability was then explored with regression analyzes and permutation tests (10,000 iterations). HI was treated as a continuous, rather than a categorical variable to better infer the relationship between the level of hybridization and the amount of developmental instability, while providing precision, flexibility and statistical power compared to ANOVAs [68, 69]. In this perspective, linear and quadratic regressions were conducted to find the optimal fit for data trends, comparing *F*-ratios and fit indices (AIC, AICc and BIC) for model choice. Developmental instability, measured as FA, could indeed express a variety of patterns, characterized by linearity or monotonous change towards some specific value [70], depending of the underlying mechanisms that drive it.

## Results

### Estimating measurement errors

Repeated ANOVA measurements were performed on a subsample of 36 honeybees, photographed and digitized twice. The relative contribution of measurement errors (ME) to the total variation for centroid size and shape, due to imaging and landmark digitizing steps (estimated by the mean squares (MS)), was small compared to the components of symmetric and asymmetric variation (Table 2). In particular, fluctuating asymmetry (FA), used as an estimate of developmental instability, was found significant, with *F* ratios sufficiently high to consider ME as negligible (*F* > 18 and 64, for shape and size, respectively) [71]. As a result, it was estimated that repeated measurements were unnecessary for the whole data set in order to obtain reliable and accurate estimates of FA.

**Table 2:** Analysis of variance of forewing size and shape in 36 honeybees photographed and digitized twice.

**Size (Centroid size)**	**ANOVA**					
Effect	**SS**	**df**	**%Var**.	**MS**	**Goodall’s *F***	***P***
*Individuals*	12209246.53	35	96.19%	348835.62	26.50	< 0.0001
*Sides*	6216.62	1	0.05%	6216.62	0.47	0.4965
*Individuals × sides*	460811.61	35	3.63%	13166.05	64.80	< 0.0001
*ME1*: *imaging*	14626.51	72	0.12%	203.15	11.33	< 0.0001
*ME2*: *landmark digitizing*	2581.80	144	0.02%	17.93		
**Shape (Procrustes distances)**	**Procrustes ANOVA**					
Effect	**SS ×10**^**5**^	**df**	**%Var**.	**MS ×10**^**5**^	**Goodall’s *F***	***P***
*Individuals*	13403.27	1190	88.35%	11.26	9.70	< 0.0001
*Sides*	119.44	34	0.79%	3.51	3.02	< 0.0001
*Individuals × sides*	1381.96	1190	9.11%	1.16	18.83	< 0.0001
*ME1*: *imaging*	150.97	2448	1.00%	0.06	2.63	< 0.0001
*ME2*: *landmark digitizing*	114.90	4896	0.76%	0.02		

df, degrees of freedom (in Procrustes ANOVA, df is that for conventional ANOVA multiplied by the shape dimension, i.e. 34); SS, sum of squares; MS, mean squares; %Var., percentage contributions of each variance component to the total variance, computed from SS values.

‘Individuals’: variation among individuals (i.e. symmetric component); ‘Sides’: directional asymmetry (DA), ‘individuals x sides’ interaction: fluctuating asymmetry (FA).

*F*_*Ind*._ = MS_*Ind*._ / MS_*Ind*. *× Sides*_

*F*_*Sides*_ = MS_*Sides*_ / MS_*Ind*. *× Sides*_

*F*_*Ind*. *× Sides*_ = MS_*Ind*. *× Sides*_ / MS_*ME1*_

*F*_*ME1*_ = MS_*ME1*_ / MS_*ME2*_

### Quantifying among individuals variation and asymmetry

The ANOVAs performed on the whole sample (Table 3) indicated that morphological variation among individuals (‘individuals’ effect) accounts for the largest portion of the variation in the whole sample (99.27% for size and 91.02% for shape, *P* < 0.0001). Directional asymmetry (‘sides’ effect) was found significant only for shape (*F* = 16.69, *P* < 0.0001), despite a tiny contribution to sample variability (0.79%). Fluctuating asymmetry also contributes to only 0.72% of size variation, compared to 8.51% for wing shape.

**Table 3:** Analysis of variance for both forewing size and shape of honeybee workers (*Apis mellifera*).

**Size (Centroid size)**	ANOVA					
Effect	SS	df	%Var.	MS	Goodall’s *F*	*P*
*Individuals*	21922690.35	301	99.27%	72832.86	137.46	< 0.0001
*Sides*	471.81	1	0.002%	471.81	0.89	0.3461
*Individuals × sides*	159485.47	301	0.72%	529.85		
**Shape (Procrustes coordinates)**	Procrustes ANOVA					
Effect	SS (×10^5^)	df	%Var.	MS ×10^5^	Goodall’s *F*	*P*
*Individuals*	27146.97	10234	91.02%	2.65	10.69	< 0.0001
*Sides*	140.00	34	0.47%	4.12	16.60	< 0.0001
*Individuals × sides*	2538.83	10234	8.51%	0.25		

df, degrees of freedom (in Procrustes ANOVA, df is that for conventional ANOVA multiplied by the shape dimension, i.e. 34); SS, sum of squares; MS, mean squares; %Var., percentage contributions of each variance component to the total variance, computed from SS values.

‘Individuals’: variation among individuals (i.e. symmetric component); ‘Sides’: directional asymmetry (DA), ‘individuals x sides’ interaction: fluctuating asymmetry (FA).

*F*_*Ind*._ = MS_*Ind*._ / MS_*Ind*. *× Sides*_

*F*_*Sides*_ = MS_*Sides*_ / MS_*Ind*. *× Sides*_

### Congruence between morphology and neutral genetic variation

**Fig 2:**
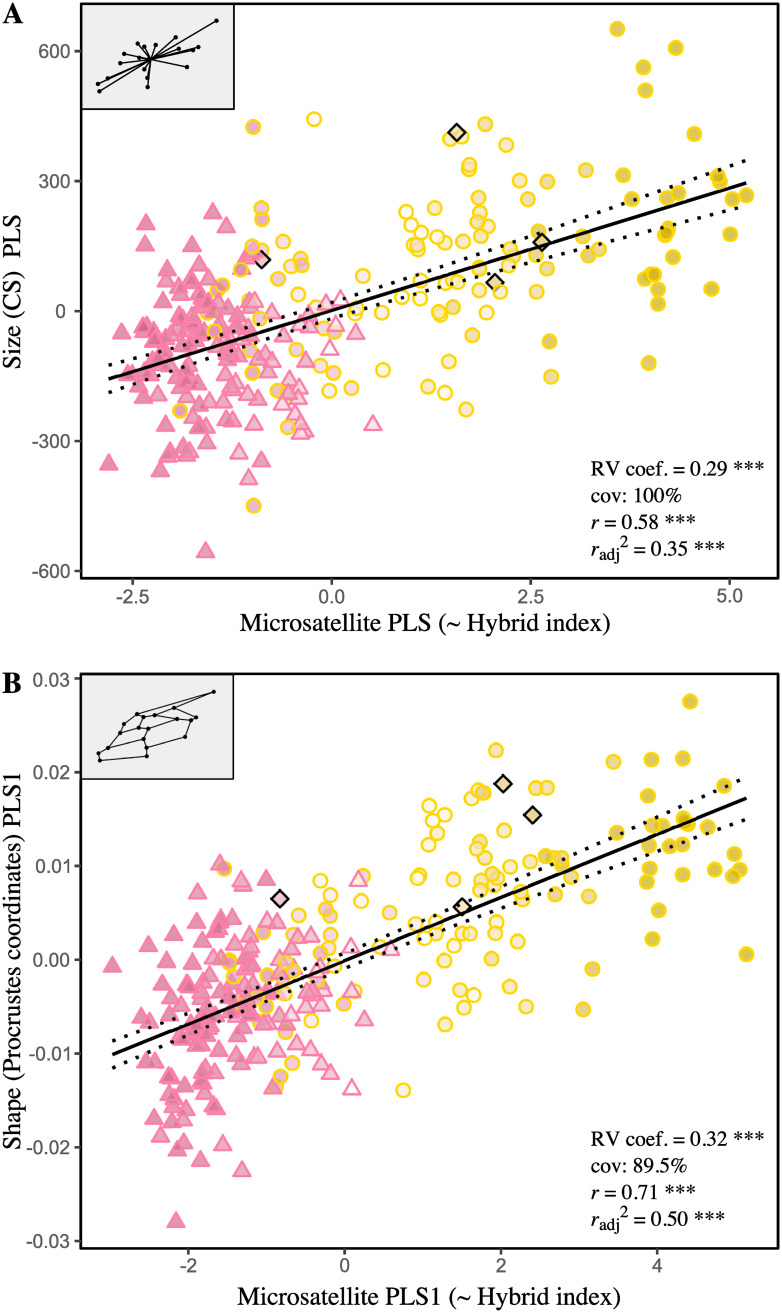
Patterns of covariation between forewing morphology and neutral genetic variation assessed *via* partial least-square analyses (PLS). (A) Scatter plot of the PLS of the forewing size (centroid size) and neutral genetic variation (14 microsatellite allele frequencies). The analysis resulted in one significant pair of PLS axes (10,000 permutations) accounting for 100% of covariation since size is a one-dimensional variable. (B) Scatter plot of the PLS of the forewing shape (Procrustes coordinates) and neutral genetic variation (14 microsatellite allele frequencies). The first pair of PLS axes is shown, which is also the only pair found significant by the permutation test (10,000 perm.). In the graphs, each point represents one individual. The symbol and outline color represents the maternal origin of individuals (pink circle: African lineage, yellow circle: European C lineage, black diamond: European M lineage). The fill color symbolizes the hybrid index (HI), i.e. the hybridization gradient from African genotypes (pink) to European genotypes (yellow). Regressions lines with 95% confidence intervals (dotted lines) are shown.

Partial least square (PLS) analyzes were used to measure the congruence of wing size (centroid size), and then wing shape variation (Procrustes coordinates), with microsatellite allele frequencies. In both cases, we found a relatively strong overall association between morphological and genetic blocks, resulting in a RV coefficient of 0.29 for size (*P*_perm_ < 0.0001) and 0.32 for shape (*P*_perm_ < 0.0001). The PLS analysis conducted between size (one dimension) and microsatellites resulted in a single pair of axes whose corresponding PLS scores are moderately and significantly correlated (SV = 220.98, *r* = 0.58, *P*_perm_ < 0.0001). Note that the size vector ordered individuals according to their wings size, so that size-PLS scores and centroid sizes are interchangeable. The PLS analysis conducted between shape and microsatellites resulted in 34 pairs of PLS axes, of which only the first was found significant, taking up 90.02% of the total covariance. Microsatellite and shape PLS1 scores were also highly correlated (SV1 = 0.014, *r* = 0.71, *P*_perm_ < 0.0001). When compared to the PCAs (S1 and S2 Figs) computed separately on the same data, the major shape and genetic PLS axes appear nearly identical to the corresponding first principal components (microsatellite PC1 = 23.35%, shape PC1 = 22.22%), with pairwise correlations displaying values greater than 0.98 and highly significant (*P* < 0.0001). Overall, these results suggest that the major dimension of variation between morphological and genetic blocks is equivalent to the dominant variation within the same blocks. The associated patterns illustrated in Fig 2A and 2B reveal a (essentially) one-dimensional morphogenetic continuum polarized between African and European lineages and reflecting the ongoing hybridization process. Besides, high similarities found between the hybrid index (HI) and the dominant genetic PCA and PLS axes (*r* = 0.98, *P* < 0.0001) prove multivariate analyses as accurate as the Bayesian method.

### Effects of hybridization on wing size

**Fig 3:**
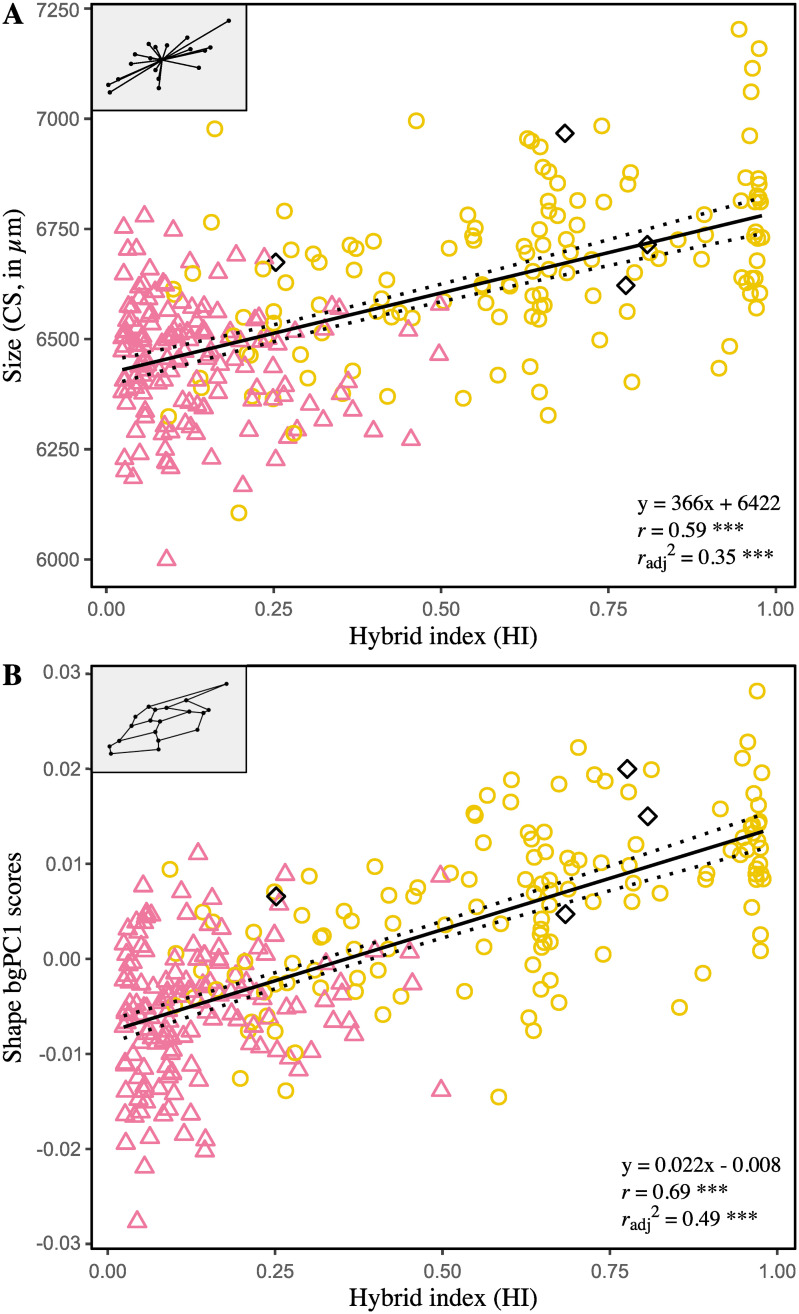
Relationships between forewing size (A) and shape (B), as captures by the between-group PCA, and the hybrid index. CS: average centroid sizes, i.e. mean of right and the left forewing computed for each individual, in μm; bgPC1: first axis of the between-group PCA conducted on individuals classified as “pure” Africans, “pure” European and hybrids. The symbol and outline color represents the maternal origin of individuals (Δ African lineage, ○ European C lineage, ◆ European M lineage) inferred from mitochondrial markers. Regressions lines with 95% confidence intervals (dotted lines) are shown.

The one-way ANOVA conducted on centroid sizes determined that differences exist among clusters, and both Tukey and Games-Howell (equal variances not assumed) post-hoc tests found highly significant differences for all pairwise comparisons of means (*P*_adj_ < 0.0001). The African cluster presents the smaller wings (6453 μm, sd = 145 μm, se = 17 μm, ci = 33 μm), the greatest ones corresponding to the European cluster (6778 μm, sd = 195 μm, se = 38 μm, ci = 77 μm), and hybrids occupying an intermediate place closer to the African cluster (6560 μm, sd = 176 μm, se = 13 μm, ci = 26 μm). Finally, as expected, wing size—treated as continuous—is positively correlated with HI (*r* = 0.59, *P* < 0.0001) and coincides well with the continuum of introgression between African and European genetic pools (Fig 3A).

### Effects of hybridization on wing shape

**Fig 4:**
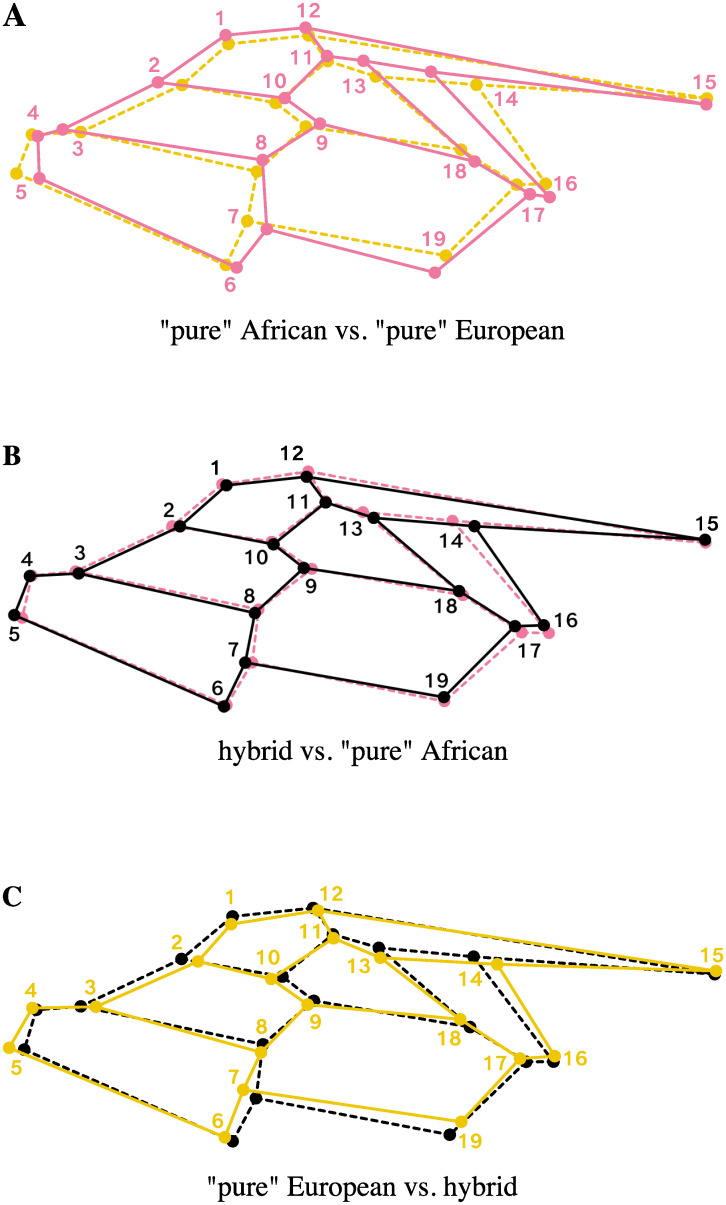
Shape changes between the means of “pure” African, “pure” European and hybrid clusters along the first axis of the between-group PCA. (A) “Pure” Africans *versus* “pure” Europeans. (B) “Pure” Africans *versus* hybrids. (C) “Pure” Europeans *versus* hybrids. Shape changes are displayed as wireframe graphs symbolizing the pattern of forewing venation of *Apis mellifera*. Pink: “pure” African mean shape; yellow: “pure” European mean shape; black: hybrid mean shape.

The between-group PCA conducted on wings shape (left-right mean of Procrustes coordinates) resulted in two orthogonal axes representing the Euclidean distances among the three cluster means. The first axis (bgPC1), accounts for 90.69% of the variation between group means, against 9.31% for bgPC2. Cross-validated classification revealed poor overall accuracy with only 57.14% of individuals correctly classified, which is largely attributable to the strong overlap of the hybrids on other clusters (correct classification rate rises to 90.20% when hybrids are excluded). ‘Pure’ Africans and ‘pure’ Europeans appear more distinct, and each presents a single misclassified individual attributed to the opposite group (‘pure’ Afr.: 72.97%, ‘pure’ Eur.: 81.48%) (Table 4). The trajectory analysis failed to find an angle statistically different from 0° between the vector connecting the mean shapes of the three clusters ($\hat{HAE}=26.13{}^{\circ}$, *P* = 0.29). Instead, it revealed significant differences of magnitudes (i.e. vectors size), suggesting intermediacy rather than transgressive phenotypes in hybrids shape with greater proximity toward ‘pure’ Africans (A—E: 0.020; A—H: 0.009; E—H: 0.013, *P* < 0.0001 for all distances). The associated shape changes as captured by bgPCA components are depicted in the form of wireframe graphs [72] in Fig 4. Notice that the joint analysis of size and shape (not shown) did not improved classification rates [61]. Again, strong association is observed between bgPC1 and HI (*r* = 0.70, *P* < 0.0001), while it is absent for bgPC2 (*r* = -0.01, *P* = 0.87) (Fig 3B).

**Table 4:** Cross-validated classification of worker honeybees as pure Africans (A), pure Europeans (E) and hybrids (H) using forewing shape.

Cluster	classified as			Correct assignments (%)
	“Pure” A	“Pure” E	Hybrids	
“Pure” A	54	1	20	72.00%
“Pure” E	1	22	4	81.48%
Hybrids	57	37	84	47.19%

### Effects of hybridization on fluctuating asymmetry

**Fig 5:**
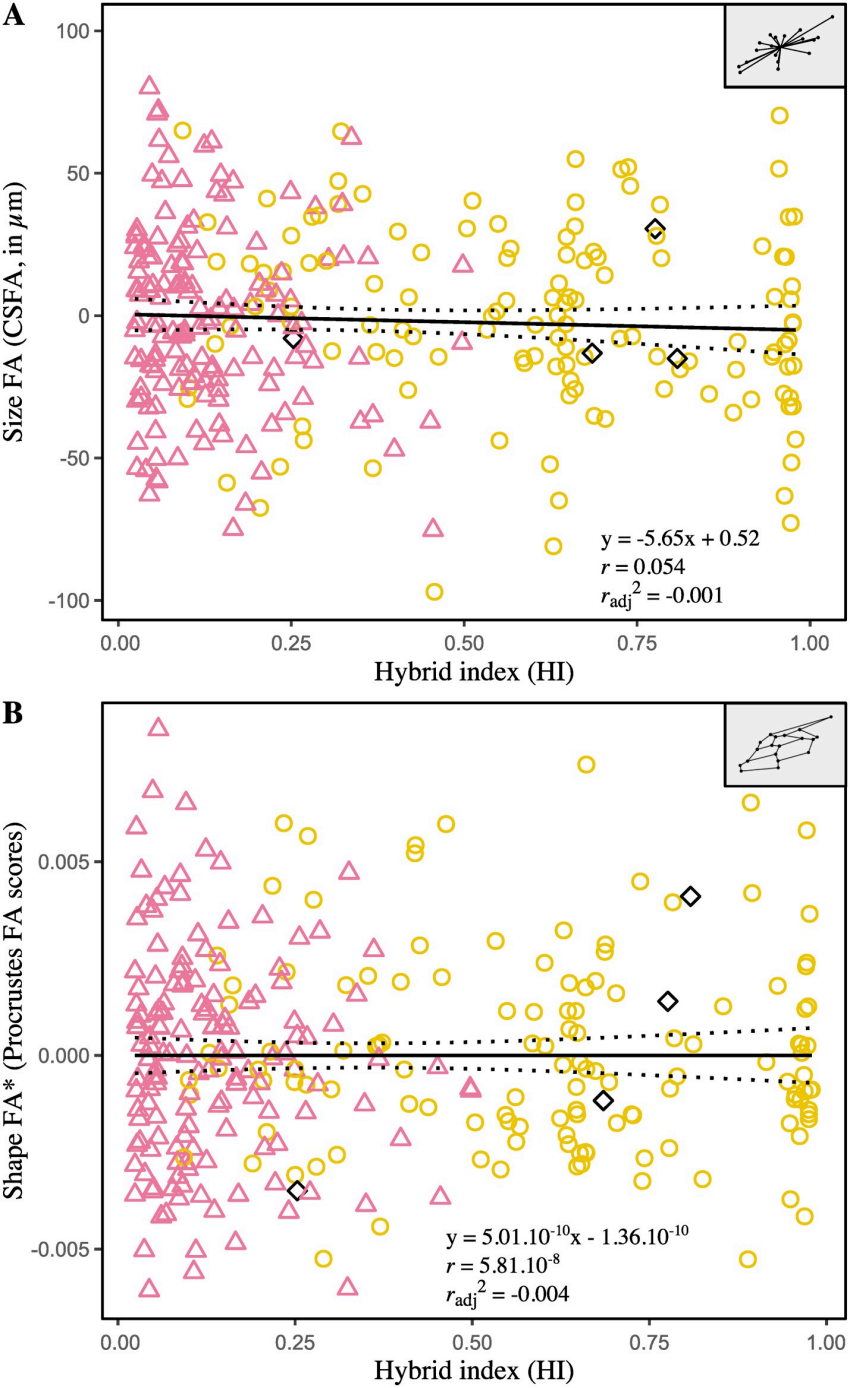
Relationships between fluctuating asymmetry of forewing size (A) and shape (B) and the hybrid index. CSFA: difference between the centroid sizes of the right and the left forewing, in μm; Procrustes FA scores: Procrustes distance between the shapes of the right and the left forewing. The asterisk (*) designates FA values corrected for size effect. Each point represents one individual. The symbol and outline color represents the maternal origin of individuals (pink circle: African lineage, yellow circle: European C lineage, black diamond: European M lineage) inferred from mitochondrial markers. Regressions lines with 95% confidence intervals (dotted lines) are shown.

Single measure intraclass correlation coefficients (2, 1) are reported to establish the reliability of univariate measures of size FA (CSFA) and shape FA (based on Procrustes or Mahalanobis distances), used to infer levels of developmental instability. Results obtained from the subsample with repeats– 36 individuals, four measurements per wing and a single examiner—indicate that repeatability is high for CSFA (0.99, 95% IC [0.987, 0.996]) and Procrustes FA scores (0.91, 95% IC [0.86, 0.95]), but low for Mahalanobis FA scores (0.02, 95% IC [-0.10, 0.19]). Consequently, Mahalanobis FA scores were excluded from the following analyzes. Linear regression analyses failed to find significant association between size and CSFA (*r* = 0.07, *P* = 0.25). On the other hand, significant effect of size on Procrustes FA scores indicates that the smaller wings tend to be more asymmetrical (*r* = -0.12, *P* = 0.04). Regression residuals were therefore used as size-corrected FA values for shape. To assess the ability of HI to predict the amount of FA, two models were constructed: one linear and one quadratic (polynomial of degree two), both compared in terms of goodness of fit. As a result, adding a quadratic term did not improve the model fits (i.e. higher values of AIC, AICc and BIC), so that the simplest (linear) has been privileged. The slopes and coefficient of determination are not significantly different from zero for both CSFA (*P* = 0.84) and size-corrected Procrustes FA scores (*P* = 1.00), indicating no association between FA and HI (Fig 5).

## Discussion

This study aims to investigate the influence of ongoing hybridization in Mauritius Island, as evidenced by genetics [40], between an initial population related to the subspecies *Apis mellifera unicolor* from Madagascar and imported European subspecies, at the phenotype level. For this purpose, individual variation and fluctuating asymmetry in forewing size and shape of honeybee workers were quantified *via* geometric morphometric methods. These traits were then confronted with neutral genetic variation to assess the effects of hybridization with exotic subspecies on phenotypic variability and developmental instability.

### Effect of hybridization with European-related subspecies on wing size and shape

Results from multivariate analyses show that morphological variation coincides well with variation in microsatellite allele frequencies. The patterns of covariance for both wings size and shape, revealed by partial least-square analyses, are (essentially) one-dimensional and parallel the level of hybridization of the individuals (Fig 2). It results in an Afro-European continuum of admixture, which also emerges in PCAs as the dominant and only intelligible pattern of variation within shape and microsatellite datasets (except unstructured phenotypic plasticity) (S1 Fig). Overall, this highlights that demographic and stochastic processes, initiated by the recent successive imports of continental European subspecies in Mauritius, are still ongoing and prevail over possible selective processes. Oleska and Tofilski [22] previously reported such agreement between morphometrics and microsatellites, but in a context of admixture between two European subspecies, *A*.*m*. *carnica* and *A*.*m*. *mellifera*. Thus, these results reaffirm the usefulness of wing geometric morphometrics for hybrid detection and to infer patterns of introgression in honeybee populations. Yet, classification of workers as pure European, pure African and hybrids show that non-introgressed individuals are still well distinct morphologically, as suggested by wings size comparisons (Fig 3A) and shape based assignment tests (Table 4; Figs 3B and 4); besides, the analyses failed to find transgressive phenotypes (i.e., exhibiting unique morphological components) in hybrid descendant. If wings size clearly reflects ancestries of the two genetic pools, i.e. smaller wings in African-related individuals and larger wings in individuals related to European commercial subspecies [19, 36, 73–77], it can only be suspected concerning wing shape, which could have been further affected by secondary evolutionary or stochastic processes [78]. Ultimately, in the absence of breeding programs and new imports—officially banned since 2011 on the island [79, 80]–the honeybee population of Mauritius should evolve towards complete admixture. Unlike the Africanized honeybee (*A*.*m*. *scutellata* × various European subspecies), which is partly characterized by a shift of European towards African traits leading notably to smaller wings and unique shapes [35], the Mauritian honeybees exhibit on average larger forewings as well as shape changes as a result of “Europeanization” of its population. A comparison with the surrounding islands and archipelagos of the SWIO where *A*.*m*. *unicolor* occurs, as well as with continental European sources, should shed light on the particular evolutionary trajectory followed by the population of Mauritius.

### Effects of hybridization with European-related subspecies on developmental instability

Developmental instability, measured as fluctuating asymmetry of bilateral traits (i.e., left-right random variations), is closely related to both the inherent genomic architecture of individuals (heterozygosity, ploidy, intra- and intergenic interactions, genomic coadaptation) and various environmental stressors, which interact in complex—and still misunderstood—ways [81]. A common assumption states that hybridization, by instigating genetic incompatibilities or breaking coadapted gene complexes, can lead to increased developmental instability in organisms; conversely, genotypes may be less able to ensure stable development under unsuitable habitats [82, 83]. In Mauritius, where individuals from separate lineages have been brought into contact and hybridize, higher levels of developmental instability were therefore expected in hybrid descendant and/or in non-native genotypes from continental European subspecies. Significant FA has been detected in the whole sample, and affects markedly more wing shape (8.51% of variation) that wing size (<1% of variation); subtle directional asymmetry was also noticed (<1% of variation), but found significant only in shape (Table 3). These results corroborate previous studies reporting the contribution of asymmetry in wing variability in honeybees [84] and other insects [64, 85, 86]. If they suggest the overall presence of stress during the development of the studied individuals, the amounts of individual size FA and shape FA, on the other hand, do not differ according to ancestry (African, European or mixed) (Fig 5). The only trend observed and already reported by Łopuch and Tofilski [87], is that individuals with larger wings tend to present less shape asymmetry. The results, therefore, do not highlight the (potential) effects of hybridization and exposure to an unusual environment solely. Intrinsic factors specific to each subspecies, bottlenecks, or a globally stressful environment are all additional factors to consider, among others, as sources of developmental instabilities that can hide or overcome differences, difficult to detect in real conditions. To date, only two publications investigated the influence of hybridization between separate lineages on wing asymmetry in *A*. *mellifera*, and only one involved both European and African lineages. The first, conducted by Smith et al. [45] on two European subspecies of M and C lineages (*A*.*m*. *mellifera* and *A*.*m*. *carnica* respectively), observed no significant effect of hybridization on wing asymmetry. The second, conducted by Schneider et al. [36], in which the African subspecies *A*.*m*. *scutellata* and a European commercial strain (combination of several European subspecies) were crossbred to mimic the first stages of the emergence of the Africanized honeybee, found lower shape FA in African honeybees relative to European and hybrid workers. In theory, the similar amounts of FA observed in the African workers compared to European (mix of *A*.*m*. *ligustica*, *A*.*m*. *carnica* and *A*.*m*. *mellifera*) and hybrids in Mauritius may result from a more sensitive developmental system inherited from *A*.*m*. *unicolor* [81], or may reflect population isolation [88]. One alternative is that other stressors interact and supplant the effects of hybridization and unsuitable habitat. Indeed, Mauritius is highly subject to human pressures. Terrestrial ecosystems of the island have been largely transformed, sparing less than 2% of native habitats [89, 90], and the majority of land area is devoted to agriculture (53%), resulting in high degrees of human disturbances. Recent studies reported increased asymmetry in honeybees related to urbanization [91, 92], exposition to pesticides [93] and pollution rates [84]. Likewise, such anthropogenic stressors could impact the development of bees in Mauritius and deserve further research.

## Conclusion

The Mauritian honeybee is experimenting intraspecific hybridization between *A*.*m*. *unicolor*-related individuals (subspecies endemic of Madagascar) and European-related subspecies, following the latter’s imports since the 19^th^ century. The strong agreements found between wings morphology, based on wings size and shape, and neutral genetic variation (14 microsatellite markers) show that the process is still going on, and reaffirm that wings morphometry can be used for hybrid detection in honeybees. Developmental instabilities, measured by individual FA and observed in both wing size and shape, affect individuals in comparable levels whatever their origin and level of hybridization. Consequently, wing asymmetry should influence equally fitness and performance (if linked to) of the workers at the population level, and might not contribute directly to trait displacements in the population, as it is suggested for the Africanized honeybee [36]. Measuring more traits, including behavioral ones, would be necessary and could help to clarify the evolutionary mechanisms driving the population. Human-mediated hybridization has surely changed the evolutionary trajectory of the Mauritian honeybee. In the shorter term, increased genetic and phenotypic variances could have improved its ability to cope with growing anthropogenic threats, as illustrated by the recent and accidental introduction of *Varroa destructor* in Mauritius [94], a mite implicated in elevated colony mortality rates worldwide as a virus vector [95, 96]. Thus, results presented by Techer et al. [39, 40] and here can beneficiate, as a reference point before *V*. *destructor* arrival, to the management of the honeybee in Mauritius in order to preserve its ecosystem and economic roles. More widely, it could provide better understanding on the impact of intraspecific hybridization and contact with new parasites on evolutionary trajectories of populations.

## Supporting information

S1 Fig Patterns of variation within neutral genetic variation (A) and forewing shape (B) from principal component analyses (PCAs). Genetic and shape structures are inferred from microsatellite allele frequencies and Procrustes coordinates, respectively. Variables were centered but not scaled. Points represent individuals genotype (A) or forewing shape (B). The symbol and outline color represents the maternal origin of individuals (pink circle: African lineage, yellow circle: European C lineage, black diamond: European M lineage). The fill color symbolizes the hybrid index (HI), i.e. the hybridization gradient from African genotypes (pink) to European (yellow) genotypes, which is highly similar to the main genetic and shape (PC1) axes.
(EPS)

S2 Fig (EPS)
